# Capturing the impact of individual characteristics on transport accessibility and equity analysis

**DOI:** 10.1016/j.trd.2020.102473

**Published:** 2020-10

**Authors:** Malvika Dixit, Aruna Sivakumar

**Affiliations:** a Department of Transport & Planning, Faculty of Civil Engineering and Geosciences, Delft University of Technology, Stevinweg 1, 2628CN Delft, the Netherlands; b Urban Systems Lab, Centre for Transport Studies, Department of Civil and Environmental Engineering, Imperial College London, Exhibition Road, London SW7 2AZ, UK

**Keywords:** Accessibility, Transport equity, Logsum measures, Individual characteristics, Disaggregate analysis

## Abstract

Transport accessibility experienced by an individual depends on their needs and abilities, as represented by their individual characteristics, such as age, income and gender. Although important from an equity perspective, the individual component of accessibility is currently ignored in most transport equity studies. This paper evaluates the impact of including individual characteristics into logsum-based accessibility measures for transport equity analysis.

Using data from the London Travel Demand Survey (LTDS) 2011–13, two alternate logsum measures of accessibility are specified – with and without individual characteristics. An empirical analysis of spatial, social and economic equity is conducted using both the measures, and the outcomes are compared. The results clearly demonstrate that ignoring individual characteristics in logsum measures of accessibility can lead to unreliable outcomes for social and economic equity analysis, but do not add significant value when aggregated across large geographical zones for spatial equity analysis. Overall, ignoring individual characteristics masks the disparity in distribution of accessibility, as measured by the Gini index. Although not straightforward, the difference between accessibility patterns using the two logsum measures also yields insights into the possible causes of inequity, which can provide actionable inputs to policy makers. The study highlights that personal needs and abilities are often responsible for accessibility variations among individuals and ignoring them can result in a misleading picture of equity, as demonstrated quantitatively in this paper.

## Highlights

• Use logsum to assess impact of individual characteristics on transport equity & accessibility.
• Alternate formulations of logsum with and without individual characteristics are compared.
• Ignoring individual characteristics leads to an underestimation of variability in accessibility.
• Individual characteristics in logsum measures provide more insight into the causes of inequity.

## 1 Introduction

Accessibility is a popular way of measuring transport performance, and is essentially the ultimate aim of most transportation activities (Litman, 2002). Accordingly, it has been widely used for measuring equity impacts of transport policies (Lucas et al., 2016). Transport equity refers to the fair distribution of impacts of any transport project or policy. Poor transport access is often the cause of social exclusion and has implications for other policy areas. A report by the Social Exclusion Unit (2003) in the UK formally acknowledged the link between transport, land-use and social exclusion, and directed local authorities to include accessibility planning in their local transport plans, and to carry out structured accessibility assessments.

The term ‘accessibility’ in this case describes the degree of connectivity to basic services, such as schools, hospitals, employment opportunities and shops, as enabled by the transport system. Ideally, an accessibility measure should be sensitive to four features: changes in the transport system (transport component); changes in land-use (land-use component); temporal constraints to transport and opportunities (temporal component); and needs, abilities and opportunities of individuals (individual component) (Geurs and van Wee, 2004). In the context of transport equity analysis, the individual component becomes particularly relevant, as the level of access experienced by a person can be strongly influenced by his/her needs and abilities. For example, given the same transport and land-use system, a low income person may not experience the same accessibility as a high income person due to differences in affordability and time budget constraints. Accessibility measures that do not incorporate personal needs and abilities fail to capture such details that are especially relevant from an equity perspective.

Accessibility can be computed at an aggregate (geographical area/zone, place-based) or disaggregate (household/individual, person-based) level. Although any accessibility measure can be calculated at a disaggregate level if sufficient data is available, not every measure can incorporate accessibility differences based on personal needs and abilities. For example, a person-level measure that is insensitive to individual characteristics such as age will erroneously assume the same level of accessibility for a young person and a senior citizen living in the same household. We therefore argue, that it is vital, from the perspective of equitable urban and transport planning, to have a measure of accessibility that allows for (i) aggregation based on individual characteristics such as income, age and gender, and (ii) the incorporation of accessibility differences arising due to these individual characteristics.

Although important for equity, incorporating accessibility differences resulting from individual characteristics can be challenging, due to which most existing studies do not include them. In addition to requiring detailed data on the needs and abilities specific to individuals, a suitable formulation is needed that can incorporate such details. The logsum is a type of disaggregate measure that is capable of capturing the impact of land-use, transport and individual characteristics on accessibility (Geurs et al., 2010). Despite its popularity in accessibility evaluations, only a few studies have used logsum for transport equity analysis (for example Bills and Walker, 2017). While all applications of the logsum measure could (in theory) be aggregated by individual characteristics, only some (such as Niemeier, 1997, and Bills and Walker, 2017) consider accessibility differences arising due to the inclusion of *individual characteristics* into the logsum measure. The inclusion of individual characteristics is typically based on the availability of data, with many studies deriving the logsums from existing travel demand models, where data on individual characteristics may be scarce. However, no study so far has explicitly evaluated the suitability of these logsums for transport equity analysis, and the extent to which the inclusion of individual characteristics affects the results of the equity analysis.

This paper explores the application of the logsum measure of accessibility to transport equity analysis, with a specific focus on determining the impact of including *individual characteristics* into the logsum measure on equity outcomes. This is undertaken by comparing two alternative formulations of logsum measures of accessibility – with and without individual characteristics, based on the same underlying data. An empirical analysis of spatial, social and economic equity is conducted using London as a case study. The accessibility contribution by each mode is computed using the compensating variation approach as proposed by Niemeier (1997), and further applied in Handy and Niemeier (1997). Since the proposed logsum measures incorporate both transport and land-use components of accessibility, they can be used to undertake equity evaluation of policies impacting both these dimensions. Moreover, if individual characteristics are included, the distributive impact of policies affecting a specific population segment can be evaluated, while taking into account their differing needs and abilities. To demonstrate this, four hypothetical policy scenarios are implemented, and their impact on various socio-demographic segments is compared using the two logsum measures of accessibility.

If the ultimate aim of equity analysis is to reduce transport inequities, it is important not only to know the existing equity patterns but also to identify the reasons behind those patterns. We have attempted to do this by disentangling the impact of individual characteristics, such as income and age, on accessibility. The aim of this research is not to introduce a new measure of accessibility, but rather to demonstrate how existing disaggregate measures can be applied and to explore the implications of different formulations for transport equity analysis.

## 2 Background

### 2.1 Equity & accessibility

**Fig. 1:**
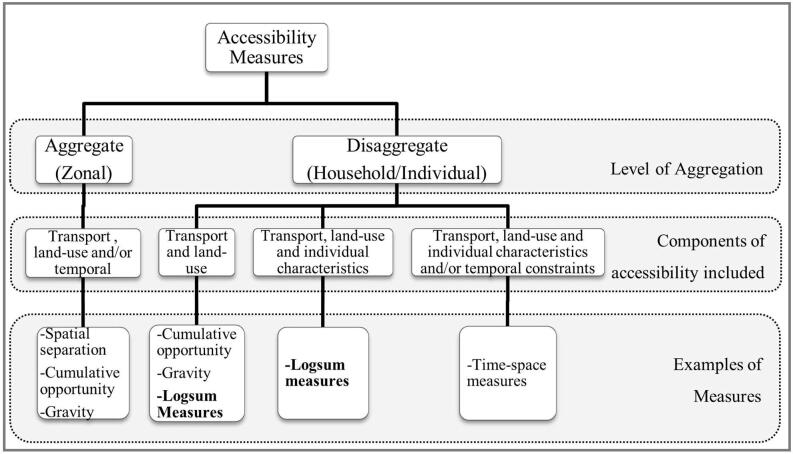
Types of accessibility measures by level of aggregation, and components.

There are various aspects to be considered when discussing transport equity analysis, and a very useful overview of the different issues and considerations can be found in Litman (2002) and Geurs and Ritsema van Eck (2001). Accessibility is one of the popular indicators used for measuring transport equity, other equity indicators include travel times, travel distance, mode share, environmental quality, project investment proximity, congested vehicle miles travelled and displacement (Bills and Walker, 2017). One of the oldest definitions of accessibility can be found in Hansen (1959), who defines it as ‘the potential of opportunities for interaction’. Following that, many other interpretations and definitions have been added (see Burns, 1979; Ben-Akiva and Lerman, 1979; Bhat et al., 2002). A variety of accessibility measures are found in the literature, ranging from a simple count of available opportunities to the more sophisticated measures that take into account the activity participation schedules of an individual. Bhat et al. (2000) classify the measures into five categories - graph and spatial separation measures, cumulative opportunities models, gravity type models, logsum/utility models, and time-space models. In this research we differentiate between aggregate (zonal) and disaggregate (household/individual) level measurement of accessibility. Although all the accessibility measures can be calculated at the desired level of aggregation, not all measures can capture the different components of accessibility, as illustrated in Fig. 1. Measures such as spatial separation and cumulative opportunities, that are sensitive only to land-use and transport components of accessibility, are typically calculated at an aggregate (zonal) level.

A disaggregate analysis of accessibility can help capture the arguably minor differences in accessibility that cannot be identified by aggregate level measures, and there is accordingly a trend towards disaggregate measures in the academic literature (Geurs and van Wee, 2004), specifically when measuring transit equity (Cao et al., 2019; Carleton and Porter, 2018; Fransen and Farber, 2019). When looking at equity impacts of accessibility, most researchers agree that the choice of measure has an impact on the conclusion derived from the equity analysis (Litman, 2002; Pritchard et al., 2019; Talen and Anselin, 1998).

A notable example comparing different measures is found in Neutens et al. (2010), who compare ten aggregate and disaggregate accessibility measures for equity analysis of access to public service delivery. The equity analysis is conducted using Gini coefficients and Lorenz curves, and by observing the differences in accessibility by gender using each measure. The measures compared include cumulative opportunity, gravity, time-space and utility-based measures. They conclude that between aggregate and disaggregate measures, the latter are more appropriate for equity analysis as they capture the accessibility variations better and provide a more inequitable picture of accessibility. While they use socio-demographic segments to compare the accessibility values, none of the measures incorporate the impact of individual characteristics on accessibility. Hence, the variations observed cannot be attributed to individual characteristics.

LaMondia et al. (2011) also compare cumulative opportunity, gravity and utility based measures for healthcare accessibility using para-transit. The utility based measure used is derived using micro simulation, and unlike Neutens et al., it considers gender and mobility requirements of an individual in the calculation of their accessibility. The study concludes that utility-based measures that can capture an individual’s perception present a more equitable picture of accessibility whereas the aggregate measures provide more extreme results. Both Neutens et al. and LaMondia et al. use utility-based measures for equity analysis but arrive at contradictory conclusions regarding equity analysis. Since one of the studies uses utility based measures that include individual characteristics whereas the other does not, and the empirical context of the studies is very different, it is not possible to draw conclusions as to reason for the differences.

van Wee and Geurs (2011) specifically identify the need to evaluate the impact of different components of accessibility on the distributional effects of policies and social exclusion. The current study takes up this task of looking at the impact of the individual component of accessibility on equity evaluation.

### 2.2 Logsum measure of accessibility

Despite a large body of literature providing theoretical as well as empirical comparisons of existing accessibility measures (see Bhat et al., 2002; Song, 1996; Baradaran and Ramjerdi, 2001; Guy, 1983; Kwan, 1998; LaMondia et al., 2011; Pritchard et al., 2019), there is no universal agreement on a single best measure. The common conclusion from these studies is that the choice of measure significantly impacts the results of accessibility analysis, and can lead to different issues being highlighted. Kwan (1998) emphasizes that an accessibility measure should be so chosen that it is able to highlight the specific variations that are required for the purpose of the study. Since this study aims to examine the impact of individual characteristics on accessibility and the implications for transport and land-use planning policy, we use the logsum measure for our analyses.

Ben-Akiva and Lerman (1979) in their paper on discrete choice models of travel behaviour, present the conceptual basis for logsum measures. Given that an individual will select the alternative which maximizes his/her utility and utility cannot be known with certainty, accessibility is made deterministic by taking the expected value of the maximum utility in a choice model, as shown in Eq. (1). That is,

(1) $${\mathrm{Accessibility}=E[Max}_{i\in C_{t}}U_{\mathrm{it}}]$$

where *U_it_* is the utility derived by an individual *t* from an alternative *i*, belonging to the choice set *C_t_*.

For the multinomial logit form of choice model, the expression for accessibility can be derived in a closed form, and it is the most common form of utility based measure. The utility for a multinomial logit model is given by

(2) $$U_{\mathrm{it}}=V_{\mathrm{it}}+\varepsilon_{\mathrm{it}}$$

where

$V_{\mathrm{it}}$ is the observable component of utility, and
*ε_it_* is the unobserved component, assumed to be identically and independently distributed across alternatives with a Gumbel distribution.

The expression for accessibility in this case is given by the log of the denominator of the choice model (Ben-Akiva and Lerman, 1985), also known as the ‘logsum’ as shown in Eq. (3):

(3) $$E\left[MaxU_{t}\right]=ln\sum_{i\in C_{t}}e^{\mu V_{\mathrm{it}}}$$

where, *μ* is the scale parameter which is related to the unobserved component *ε* as Var[ε] = π^2^/6μ^2^. It is normalized^1^ to 1 here for simplicity.

This measure provides a summary of the maximum utility of all travel alternatives available to an individual (Ben-Akiva and Lerman, 1985), effectively combining multimodal accessibility into one single measure, and reflecting the benefit of choice (in modes and destinations) available to individuals. The log form captures the decreasing marginal utility of the alternatives. One of the key advantages of the logsum measure is its consistency with random utility theory and the fact that it can be extracted from an existing travel demand model used for forecasting with little additional computation (Ben-Akiva and Lerman, 1977). The relative importance of the different components of utility included is derived based on actual travel behavior, as opposed to the cumulative opportunity or gravity based measures that assume a trade-off between the destination attractiveness and travel impedance (Handy and Niemeier, 1997). Geurs and van Wee (2004) note that the logsum measures in use today satisfy most requirements of an ideal accessibility measure except the temporal criteria.

A notable empirical study is that of Niemeier (1997) who uses logsum measures based on MNL mode-destination choice models for the Puget Sound Region in Washington. She proposes a consumer welfare approach by converting the change in accessibility to monetary values for easy interpretation and comparison, and uses this approach to compare accessibility by income categories. Some other examples of empirical studies using logsum measures are found in Sweet (1997), LaMondia et al, (2011), Geurs and Ritsema van Eck (2001) and Bills and Walker (2017).

Although logsum measures are capable of incorporating individual characteristics, such an application has significant data requirements. Based on the extent of data available and the level of detail required, the logsum measures can (theoretically) include the impact of any number of individual characteristics. Niemeier (1997) included gender, income and type of job to segment mode and destination attributes. Geurs and Ritsema van Eck (2001) on the other hand include income as the only individual attribute impacting accessibility to employment in the Netherlands. Another application by Bills and Walker (2017) also includes income as the only individual attribute in their logsum accessibility measure formulated based on an activity-based mode choice model of the Bay area.

Logically, the inclusion of each additional individual characteristic in the logsum measure should be able to highlight the accessibility variations specific to that individual characteristic. It should then be possible to segregate the impact of individual characteristics on accessibility by looking at the difference between the measures with and without the specific individual characteristics. Such an analysis can explicitly compute the impact of the individual characteristics on the logsum-based measure of accessibility. However, this has not been attempted as yet.

## 3 Data and methods

To evaluate the performance of alternate specifications of logsum measures from an equity perspective, an empirical analysis is conducted using London as a case study. One of the primary reasons for selecting London is the availability of rich travel survey data with the necessary details about individual characteristics. The highly heterogeneous zones, and the diversity of population make the city especially interesting from an equity perspective. Additionally, the extensive modal choice available with a fairly uniform modal split make it appropriate for testing various transport and land-use related policies.

Equity analysis can be conducted for a variety of different travel purposes. The constraints inherent in school/hospital ‘choices’ due to the UK policy of catchment areas makes it difficult to represent this behavior through a choice model. On the other hand, work travel has no such constraints, and usually comprises fixed travel patterns. It is a mandatory trip for most people, making it important from an equity perspective. Therefore, this study undertakes an equity analysis for accessibility to work in London.

For this, we first develop two logsum measures of accessibility based on mode-destination choice models with (Model 2) and without (Model 1) individual characteristics. The rest of this section presents three components of our research: Section 3.1 describes the data sources employed and the data preparation tasks, Section 3.2 presents the mode-destination choice model formulations (Model 1 and Model 2) from which the logsum measures are derived, and Section 3.3 describes the compensating variation approach to comparing the logsum measures in equity analysis.

### 3.1 Data sources and data preparation

The travel behavior data used for developing the logsum measures is sourced from the London Travel Demand Survey (LTDS). As part of the LTDS, about 8000 households are interviewed each year, and a one day travel diary for each person above the age of five is collected. Additionally, information regarding socio-demographics and usual patterns of travel to school/work is requested. To ensure a reasonably sized sample, two years (2011–12 and 2012–13) from the LTDS have been chosen for the analysis. It is assumed that the transport and land-use would not have changed significantly during this period, as supported by a Transport for London report (2019).

For this research, the chosen destination and mode of travel to work is determined based on an analysis of the usual travel patterns, instead of using the travel dairy data for the survey day. This ensures that we maximise the use of the data; and we avoid biasing the accessibility measure by considering a travel mode that may have been atypical for reasons specific to the diary day. The LTDS classifies the main mode of travel to work into 16 categories. These modes were aggregated into four broad categories for the analysis –

▪ Non-motorised modes: including walk and cycle,
▪ Car (including motorbike and car, as both driver and passenger),
▪ Bus, and
▪ Train (including London underground, National Rail, DLR and Overground).

Bus and train modes are treated separately due to differences in travel cost and density of network for the two modes.

Persons in part-time employment were excluded from the analysis, as they are believed to have relatively greater spatial-temporal constraints that are difficult to capture with the available data. Furthermore, persons with household or work location outside the London boundary, and those for whom mode choice information is not available have also been excluded from the sample. A final sample size of 8653 persons was therefore obtained for the analysis. Table 1 presents the socio-demographic details of the sample.

**Table 1:** Socio demographic details of sample.

Category	Segment	Number of individuals
Gender	Male	4625 (53.5%)
	Female	4028 (46.6%)
Age	<40 years	4874 (56.3%)
	40–60 years	3381 (39.1%)
	>60 years	398 (4.6%)
Monthly income per person in the household	<£1000	2809 (32.5%)
	£1000–£2000	3075 (35.5%)
	>£2000	2769 (32.0%)

**Fig. 2:**
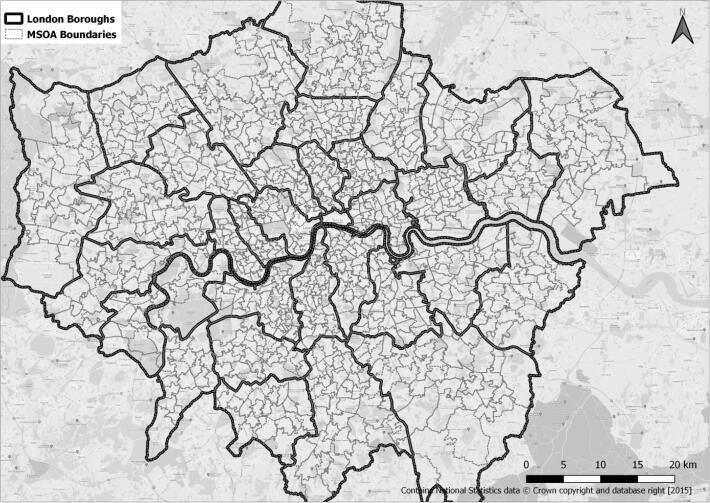
Study area, MSOAs and London boroughs.

The work locations for these individuals are defined at a middle layer super output area (MSOA) level. MSOAs are created by aggregating census output areas and are a standard census unit for representing local statistics. An MSOA typically consists of 2000–6000 households. For this study, the 2011 MSOA definition was used; this divides the Greater London area into 983 MSOAs. Fig. 2 shows the map of the study area split by MSOAs and London boroughs.

For each individual in the dataset, along with the chosen alternative identified from the LTDS, travel time information for all available mode-destination combinations is required. These travel times were estimated based on road and transit networks using a GIS software, and an accessibility planning tool ‘Visography TRACC’ which can generate multimodal travel time matrices.^2^ For this process, the residential location of the individual was approximated as the population weighted centroid of the residence MSOA, and all travel time and cost estimates were calculated at an MSOA to MSOA level. Travel time estimations for 983 × 983 × 4 origin-destination-mode combinations was thus obtained.

Not all mode-destination alternatives are available to all individuals. Hence, an availability matrix was created for each mode, based on the following assumptions: Non-motorized mode is not available for destinations where travel time is greater than 120 min (based on the maximum duration of non-motorized trips observed in the LTDS); Train is not available for within zone travel; and Car is not available to persons who report that they do not have any vehicle at their disposal.

For the calculation of travel costs by transit modes, estimates based on the monthly travel card cost between origin and destination fare zones was used (approximated as the farezone of the train/underground station nearest to the MSOA centroid). This cost was divided by the average number of working days in a month to arrive at the cost per day, and further divided by two to get the cost per trip. Travel cost by car consists of the congestion charge only. An assumption of zero congestion charge was made if both home and work locations are outside the congestion charging zone. While not entirely accurate, as individuals could drive through the congestion charging zone even if their home and work locations are outside the zone, this assumption is expected to be reasonable considering the presence of ring roads surrounding the congestion charging zone. Appropriate discounts were applied for all travel costs. In case of transit, this means free travel for disabled and older people (>60 years). For the congestion charge, discounts were applied for residents inside the congestion charging zone, and no charge was applied to disabled persons, and persons using motorbikes.

To obtain ‘destination attractiveness’ for work travel, the number of people working in an MSOA was used as a proxy for the number of work opportunities available there. The number of people working in each MSOA was obtained from Census, which groups it by the ‘National Statistics Socio-economic Classification’ (NS-SEC). This classification is derived based on both occupation and employment status (see Office for National Statistics (2010) for more information). For each individual in the LTDS data, the number of jobs available in each MSOA were tailored based on his/her occupation type (8 categories). For this, the occupation codes from LTDS were matched with NS-SEC codes based on the guidelines from the Office for National Statistics (2010).

### 3.2 Mode-destination choice model formulations

The observable component of utility *V_it_* in the logsum formulation (as shown in Equation (2)) can be expressed as a function of the attributes of the alternatives, typically modes and destinations. That is,

(4) $$V_{\mathrm{it}}^{1}=f\left(M_{i},D_{i}\right)$$

where

$V_{\mathrm{it}}^{1}$ is the observable (deterministic) component of utility corresponding to the formulation of Model 1 (i.e. without individual characteristics),
*M_t_* is the vector of modal attributes, and
*D_t_* is the vector of destination specific attributes.

The utility formulation in Equation (4) assumes that the values people place on different components of utility is the same across the population. To incorporate heterogeneity in this formulation, the value placed on the different components of utility (*M_t_* and *D_t_* in Equation (4)) can be segmented based on individual attributes *I_t_* (Equation (5)). For example, a modal attribute of travel cost may be segmented by an individual’s income level to incorporate the differences in sensitivity to travel cost based on income. The observable utility in this case (including individual characteristics) is given by

(5) $$V_{\mathrm{it}}^{2}=f\left(M_{i},D_{i},I_{t},\right)$$

where $V_{\mathrm{it}}^{2}$ is the observable component of utility corresponding to the formulation of Model 2, i.e. including individual characteristics, and *I_t_* represents the vector of individual attributes like age, gender, income, etc.

Table 2 lists the attributes of alternatives and individuals that were used in these model formulations. To ensure that the zonal structure of the study area does not affect choice probabilities, the size variable (number of jobs in each MSOA) enters the utility function in log form (Daly, 1982).

**Table 2:** Attributes used for model development.

Accessibility Component	Attribute
Land-use	Number of jobs by occupation type (Office for National Statistics, 2010): ▪ Higher managerial, administrative and professional occupations ▪ Lower managerial, administrative and professional occupations ▪ Intermediate occupations ▪ Small employers and own account workers ▪ Lower supervisory and technical occupations ▪ Semi-routine occupations ▪ Routine occupations* ▪ Never worked and long-term unemployed
	Location of destination (in terms of London fare zone with zone 1 corresponding to central London)
	Location of origin (in terms of London fare zone)
Transport	Travel time by each mode
	Travel costs by each mode
	Travel distance
Individual characteristics	Age
	Gender
	Income
	Occupation
	Number of children (<5 years)

* ‘Routine’ occupations correspond to the ones regulated by a basic labor contract.

The indirect utility functions were specified using the attributes in Table 2 for each of the 983 × 4 alternatives (mode-destination combinations), and the chosen mode-destination combination was obtained from LTDS data. Several different specifications for Model 1 and 2 were tested using the Biogeme software (Bierlaire, 2003). Final models used for equity analysis were shortlisted based on the log-likelihood ratio-based model fit statistics, the statistical significance of the parameters, and the appropriateness of the attributes for equity analysis. The selected models are presented in Table 3 with their estimation results.

**Table 3:** Estimated parameters for the two mode-destination choice models.

Description of parameter	Model 1 (without individual characteristics)		Model 2 (with individual characteristics)	
	value	p-value	value	p-value
Alternate specific constant for car	0.00	<fixed>	0.00	<fixed>
Persons in routine or semi-routine occupations using car mode			0.884	0.00
Alternate specific constant for bus	−0.585	0.00	−0.828	0.00
Females using bus mode			0.459	0.00
Alternate specific constant for train	−2.42	0.00	−1.98	0.00
Old persons (>60 years) using train mode			−1.32	0.00
Alternate specific constant for walk	−1.18	0.00	−0.981	0.00
Destination attractiveness (log of number of job opportunities by occupation type)	2.40	0.00	2.42	0.00
Travel time by bus	−0.0655	0.00	−0.0691	0.00
Travel time by bus for persons from low income households			0.0070	0.00
Travel time by train	−0.0254	0.00	−0.0232	0.00
Travel time by car	−0.0931	0.00	−0.0984	0.00
Travel time by walk	−0.0641	0.00	−0.0636	0.00
Travel time by walk for females with children (<5 years) in the house			−0.0296	0.00
Travel cost by bus	−0.340	0.00	−0.204	**0.03**
Travel cost by train	−0.109	0.00	−0.230	0.00
Travel cost by train for persons from low income households*			−0.209	0.00
Travel distance by car	−0.172	0.00	−0.168	0.00
Congestion charge, specific to the car mode	−0.0732	0.00	−0.0742	0.00
Destinations located in zone 1 defined for train mode	0.531	0.00	0.719	0.00
Destinations located in zone 1 defined for car mode	−1.25	0.00	−1.23	0.00
Destinations within 15 min walk defined for walk mode	1.24	0.00	1.23	0.00
**Final log-likelihood**	**−44,412.48**		**−44,181.09**	
**Adjusted rho-square**	**0.280**		**0.283**	
**AIC**	**88,854.96**		**88,404.17**	
**BIC**	**89,094.90**		**88,740.09**	

* Low income household is defined as having an income per person (household income/number of persons in the household) of less than £1000 per month.

Only parameters that were significant at the 99% confidence level were retained in the models, except the parameter for travel cost by bus, which is significant at the 95% confidence level. The model fit, as described by the adjusted rho square, AIC and BIC, are all marginally better for Model 2 than Model 1.

Some interesting observations emerge when incorporating the effects of individual characteristics. There is a preference amongst women (compared to men) towards the use of bus, and amongst persons employed in routine or semi-routine jobs (compared to other occupation types) towards the use of car, as shown by the high positive values of their respective parameters. It is also noted that persons over the age of 60 have a dislike towards travel by train. Possible explanations for this might include the lack of step-free access to platforms at many train stations (especially in the London underground), the discomfort experienced because of crowding in the trains or during boarding/alighting. The coefficients for travel time by bus and travel cost by train have been segmented by income and the results are as expected – the sensitivity to travel cost by train is much higher for low income persons (compared to middle and high income persons), and for travel time by bus their sensitivity is slightly lower. We also find that females who have children (<5 years) in their household are more sensitive to travel time by walk.

### 3.3 Compensating variation approach

Having identified the best specifications for models 1 and 2, the logsum measures of accessibility corresponding to each model formulation were calculated (measure 1 and measure 2, respectively). However, the absolute values of logsum measures are meaningful only if a benchmark value of accessibility is established (Ben-Akiva and Lerman, 1985). Hence, instead of comparing the absolute values, the difference in accessibility levels across two scenarios was compared, which is also interpreted as a measure of consumer surplus (Williams, 1977). For example, the accessibility by each mode for each of the logsum measures was obtained by implementing a hypothetical scenario of removing one (or more) modes. Then the difference in logsums between the base scenario and removal of mode gives the accessibility contributed by the removed mode. This is a fairly typical approach for computing and comparing the impacts of specific modes on the logsum accessibility measure (see for example, Niemeier, 1997).

The change in accessibility (due to the ‘removed’ mode) was then converted to monetary or travel time units using the compensating variation approach – as proposed by Small and Rosen (1981), and used by Niemeier (1997) and Xu (2012) among others. Compensating variation can be interpreted as the amount a person needs to be compensated in order to make them as well-off as before the policy change (Freeman III et al., 2014).

Hence, the accessibility for an individual ‘*t’*, derived from the compensating variation in travel time (or cost) units is given as in Eq. (6)

(6) $$A_{t}=\left(\frac{1}{\alpha_{t}}\right)\left[\ln\left(\sum_{k}e^{V_{\mathrm{tk}}^{1}}\right)-\ln\left(\sum_{k}e^{V_{\mathrm{tk}}^{0}}\right)\right]$$

where α_t_ is the associated travel time (or cost) parameter. The superscripts 0 and 1 correspond to the scenarios before and after the policy change, respectively.

For this study, an aggregated travel time parameter across all modes, but specific to each model formulation, was used. This was obtained by estimating additional models with generic travel time coefficients.

## 4 Equity analysis and discussion

Next, we use the two logsum measures (measure 1 from Model 1, and measure 2 from Model 2) for equity assessment. The aim of this exercise is two-fold – (i) to explore the strengths and weaknesses of the two logsum measures for equity analysis, and (ii) to investigate the causes of observed equity patterns using the differences in the two measures. To this end, we first undertake spatial (Section 4.1), social and economic (Section 4.2) equity analysis by comparing the accessibility distributions geographically, and between different social and economic segments of the population. Then we compare overall equality levels, as summarized by Gini coefficients (Section 4.3). Lastly, we use the two logsum measures for policy analysis (Section 4.4).

### 4.1 Spatial equity

**Fig. 3:**
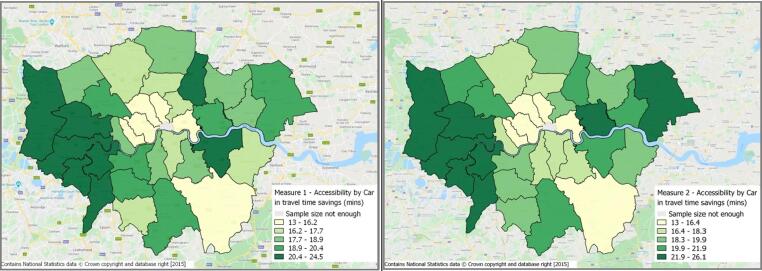
Accessibility by car for London boroughs using measures 1 and 2.

Spatial equity trends using the two logsum measures are compared for car and public transport modes. The spatial comparison was undertaken at the level of the London boroughs (instead of MSOAs) to ensure a reasonable sample size per unit. The average accessibility across all (sampled) individuals in the borough was used for this. Fig. 3 presents the spatial distribution of accessibility levels by car as calculated by the two measures. It is important to remember here that although expressed as travel time savings, the logsum measure combines the impact of travel attributes such as cost, time, distance and destination attractiveness across the various mode-destination alternatives.

At a borough level, both the measures show lower accessibility levels in central London. Although more job opportunities are located in central London, this trend is expected, as both the logsum measures incorporate (a) the congestion charge in central London, and (b) the inherent dis-preference among private car users for driving into central London, as captured by the parameter on the zone 1 dummy variable. This dis-preference among private car users may be the result of generally high levels of congestion and parking costs in Central London. Additionally, measure 2 also captures the preference for car by persons in routine and semi-routine occupations, resulting in the slight differences between the two measures (especially towards east London).

**Fig. 4:**
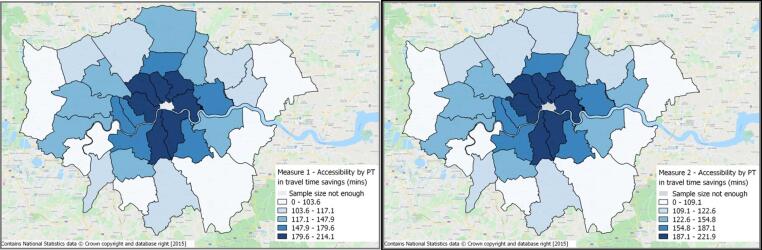
Accessibility by public transport for London boroughs using measures 1 and 2.

Accessibility by public transport (Fig. 4) is found to be higher in central London using both the measures, owing to more job opportunities, as well as a denser public transport network. In this case, the two measures are found to show very similar trends suggesting that when aggregated at a borough level, the within-zone heterogeneity gets averaged out, and inclusion of individual characteristics does not add much value.

From a policy perspective, the spatial distribution produced by the logsum measures of accessibility do not yield much insight into the observed accessibility pattern. Therefore, if the policy focus lies in accessibility evaluation across large geographical areas, like the London boroughs, there is limited value in segmenting the logsum measures by individual characteristics.

### 4.2 Social and economic equity

**Fig. 5:**
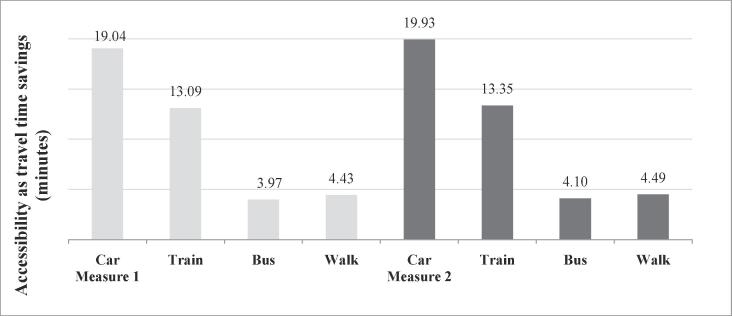
Accessibility by mode using logsum measures 1 and 2.

One of the strengths of logsum measures (which are computed for each individual in the dataset) is their ability to be aggregated across a variety of dimensions. This section considers three such dimensions - gender, age and income levels. Accessibility is compared for car, train and bus modes individually to reveal the differences specific to each mode. Before looking at the socio demographic trends, the overall accessibility levels by mode are observed (Fig. 5).

It can be seen that the two measures show very similar accessibility trends by mode. The relative accessibility levels reveal the accessibility of each mode as perceived by the individuals. The higher accessibility by car and train modes over bus and walk modes are a result of the inherent preferences people place on these modes, as well as the values placed on shorter travel times, lower travel costs, etc.

#### 4.2.1 Distribution by gender

**Fig. 6:**
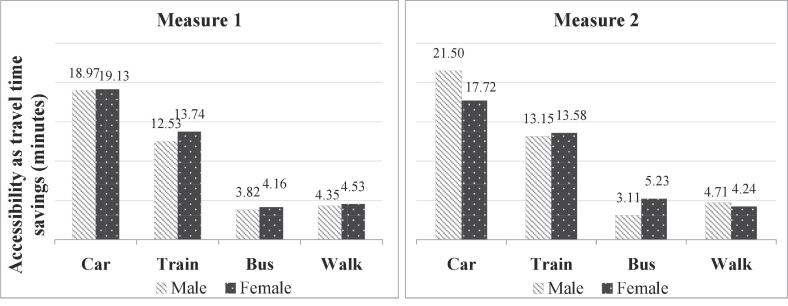
Accessibility by gender using logsum measures 1 & 2.

If individual characteristics are not considered as a determinant of accessibility, males and females are found to have similar accessibility levels for all modes (Fig. 6, measure 1). The accessibility is observed to be slightly higher for females, especially for train. However, when we incorporate the impact of gender on accessibility in measure 2, different conclusions are derived for different modes. The trends in train and walk accessibility follow the ones observed using measure 1. However, the accessibility due to car is observed to be lower for females as compared to males, whereas the accessibility due to bus is observed to be higher for females. This is explained by the additional utility that females associate with buses, as reflected by the positive parameter in Model 2 (Section 3.2). The lower accessibility due to car might be a result of a possible correlation between males and routine/semi-routine jobs. As noted during model estimation, this occupation group has a preference towards car usage.

#### 4.2.2 Distribution by income

**Fig. 7:**
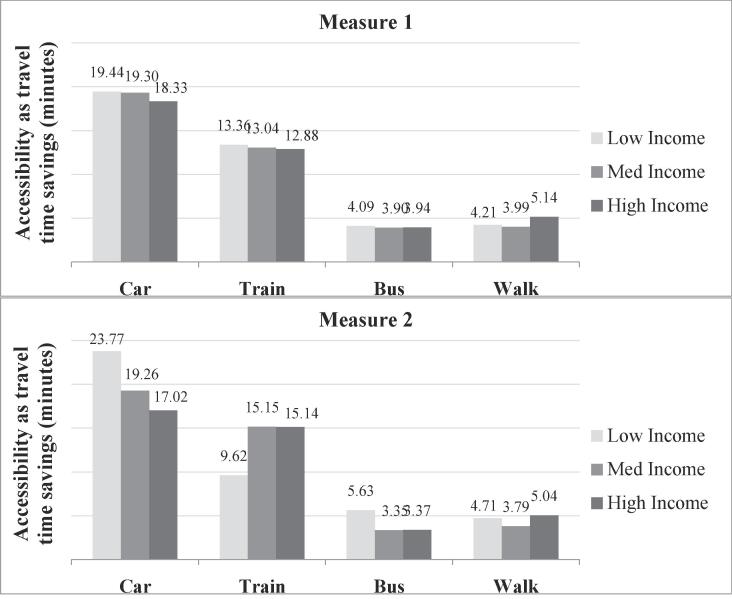
Accessibility by income using logsum measures 1 & 2.

To analyse the distribution by income, households were classified into low, medium and high income categories based on the income available per person in the household^3^. As with the gender trends, when the individual characteristics are aggregated, the three income groups seem to have similar levels of accessibility by all modes (Fig. 7, measure 1).

Accessibility computed using measure 2 reveals significant differences in accessibility based on income levels. The accessibility by car is observed to decrease with increasing income. This is explained by the fact that low income persons tend to be associated with routine and semi-routine occupations that have a relatively higher level of car accessibility. It could also mean a possible correlation between household location and income, since persons residing in zone 1 tend to avoid car usage, as noted in Section 3.2. The accessibility by train is much lower for low income persons compared to others, due to their higher cost sensitivity towards train fares. On the other hand, bus has a higher accessibility for lower income groups, as they tend to be less sensitive to bus travel time compared to other income groups, as reflected by the model parameter for it.

#### 4.2.3 Distribution by age

**Fig. 8:**
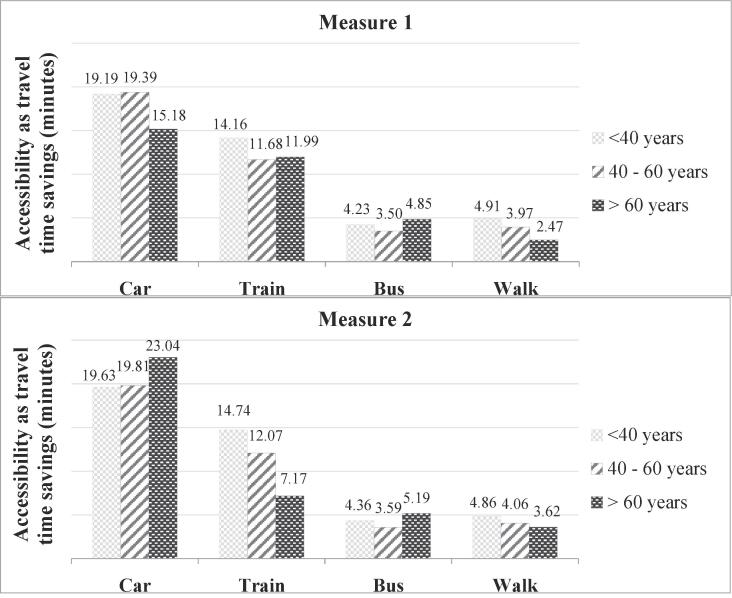
Accessibility by age using logsum measures 1 & 2.

Looking at the distribution by age, accessibility as reported using measure 1 shows different trends for different modes (Fig. 8, measure 1). Car accessibility is observed to be significantly lower for older persons. For train, the accessibility is highest for young persons (<40 years) and almost equal for the rest. Walk accessibility is observed to decline with age, as expected.

When the effect of preferences and abilities is considered, contrasting trends are observed for the car and train modes. Measure 2 incorporates the dis-preference for train mode by older persons (Section 3.2). The high accessibility for car, as observed with measure 2, for older people can be computationally explained based on the formulation of the logsum measure. Since the logarithm diminishes the marginal value of utility for each additional mode, and the accessibility by car is derived as the difference between accessibility by all modes and the accessibility on removal of the car mode, the dis-preference for train by older persons increases the value they place on the utility derived from the car mode. From a practical perspective, this potentially means that the dis-preference for train among older people is effectively a relative preference for all the other modes including car, resulting in a greater accessibility by the other modes.

#### 4.2.4 Discussion

Since logsum measures are computed at an individual level, they can be aggregated across any individual characteristic (or their combinations) and can disentangle the difference in accessibility for each socio-demographic group. Furthermore, if a logsum measure including individual characteristics is used, the power of these measures capturing accessibility differences across socio-demographic segments is further magnified.

In the social and economic equity analysis conducted, the two measures often resulted in contradictory patterns. Measure 1 presented a more uniform picture of accessibility compared to measure 2. A comparison of accessibility patterns using the two measures can also help identify the causes behind accessibility differences between population segments. For example, the difference in accessibility by train for low income individuals (using the two measures) demonstrates that although the transport supply and land use available to all income groups may be similar (as shown by measure 1), low income persons do not have the same accessibility levels as higher income persons (as shown by logsum measure 2) owing to issues such as affordability.

The choice of socio-demographic characteristics for the analysis presented in this paper is driven partly by the empirical context of the London case study and the available data. However, the analysis is intended to make a point, which it clearly does, arguing for the use of more disaggregate measures of accessibility (that account for sociodemographic differences) for equity analyses.

### 4.3 Equity insights

The logsum measure by definition is based on observed travel patterns. Accordingly, it depends on people’s underlying perception of utility, and falls under the category of ‘perceived’ measures (Morris et al., 1979). The basic difference between the two logsum measures used in this study lies in their treatment of constraints and preferences. An equitable distribution of accessibility as measured using logsum measure 1 shows up to be inequitable when measured using logsum measure 2. This reflects the fact that the same level of service provided by the transport network might not provide the same benefits to everyone. Martens and Golub (2012) provide an argument against the use of utility based measures for equity analysis in that it is unfair to provide differently for individuals with differing tastes. However, what this argument overlooks is the fact that utility based measures reflect not only individual tastes, but also their needs, abilities and opportunities - as defined by Geurs and Ritsema van Eck (2001). In the context of the current study, tailoring the number of opportunities available to an individual based on their occupation type is an example of incorporating the individual’s ‘need’. ‘Abilities’ relate to their physical capacities, such as the higher sensitivity to travel time for females with children. And ‘opportunities’ entail issues such as affordability based on one’s income. While it is true that providing for differing tastes might not be equitable, differing abilities, needs and opportunities is one of the reasons why inequalities exist in transport.

Hence, despite the misgivings of Martens and Golub, we argue that logsum measures capture the inequalities that exist due to personal characteristics, which cannot be captured by most of the other existing measures. Several researchers acknowledge that accessibility of an individual depends on the individual’s perspectives and perceptions (Curl et al., 2015), and therefore individual needs and perceptions must be included in accessibility planning (Lättman et al., 2018). In this paper, we go further to demonstrate that in using logsum measures to capture inequalities arising from differing abilities, needs and opportunities, it is important to design the logsum measures so that they explicitly incorporate sociodemographic characteristics. As otherwise they fail to appropriately capture the heterogeneities in the abilities, needs and opportunities.

#### 4.3.1 Equity computed with Gini coefficients

As observed in the previous sections, logsum measure 1 suggests that accessibility across the sub-sections of the population (males versus females, low income versus high income, etc.) has less variation, and is therefore more equitable. Here we compare the distribution of accessibility across the population as a whole using Gini coefficients. Gini coefficient (Delbosc and Currie, 2011) is a quantifiable measure of inequality^4^ with a value between 0 and 1, and is based on Lorenz curves which are a graphic representation of the distribution of cumulative income across the population (Lorenz, 1905). A value of 0 denotes perfect equality, whereas a value of 1 indicates perfect inequality, meaning that all income is concentrated with one individual.

Gini coefficients for our case study show that inequality of accessibility is very different for different modes (Table 4). Irrespective of the measure used, the distribution of accessibility by car is found to be the most equally distributed across the population. Accessibility by train and bus is more inequitable, followed by the accessibility by walk.

**Table 4:** Gini Coefficients for accessibility by mode.

Mode	Measure 1	Measure 2
Car	0.135126	0.220411
Train	0.354878	0.375843
Bus	0.358647	0.411938
Walk	0.450947	0.461751

Looking at the differences between the two measures, the higher Gini coefficients for measure 2 for each mode indicate that if individual characteristics are ignored, a more equitable picture of accessibility is obtained. This is as expected, since measure 1 overlooks the variations in accessibility based on income, gender, age and occupation, thus underestimating the inequality of accessibility within the population. The underestimation of equality is highest for car, followed by bus. From a policy perspective, if measure 1 is used instead of measure 2, the underestimation of equity impacts would be the highest for these modes.

### 4.4 Policy scenarios

Next, we evaluate the two measures for their suitability in conducting what-if policy analyses. Such an assessment can be used to compare alternate policies to select the most equitable one. Two transport and two land-use policies have been selected, to demonstrate the range of policies that can be addressed, as well as their ability to capture the impact of policies targeting a specific population segment. In selecting the policies, we were constrained by the available data and the model specifications based on the context of our empirical case study, to select policies that trigger the parameters in the mode-destination choice models presented in Section 3.2. However, the analysis presented in this section is aimed to demonstrate the strength of the logsum measures with individual characteristics (logsum measure 2) in producing more reliable equity analyses for a variety of policy scenarios. The subsequent sections present the results of implementing each of the policy scenarios, the impact of which were estimated using the compensating variation approach as described in Section 3.3. Here the policy impacts are aggregated across all modes to present the overall impact of the policies on different age, gender and income groups.

#### 4.4.1 Policy 1: An increase in total job opportunities in central London

**Fig. 9:**
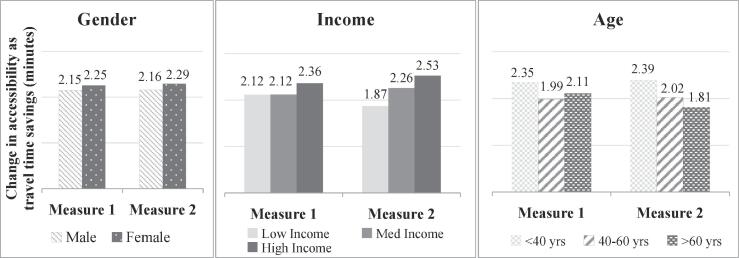
Accessibility impacts of land-use policy 1.

We start by evaluating the distributive impact on transport accessibility of increasing job opportunities by 10% in London zone 1. This represents, for example, a land use policy that invests in increased and affordable office space in zone 1 only, resulting in an increase in job opportunities. Fig. 9 shows the impact of implementing this policy on various sub-sections of the population, based on the two logsum measures.

The impact of increasing jobs in central London is found to be higher for females compared to males. There may be several reasons for this. Since the number and availability of jobs is matched based on an individual’s occupation, it is possible that the proportion of women matching with occupations found in zone 1 are higher than men. Since the trend does not change significantly between the two measures, we note that this is an aggregate impact, and not a result of individual abilities and preferences. The plot for income shows a higher impact on the high income category, whereas for age the impact is highest for young persons (<40 years). The differences in measure 1 and measure 2 show that if individual characteristics are ignored, the impact of increase in jobs in the center is slightly overestimated for low income and older age persons. Overall, the similar trends between the two measures show that the effect of individual characteristics on distributional impacts of this policy is not very significant.

#### 4.4.2 Policy 2: An increase in only senior level job opportunities

We then evaluate the equity impacts of a policy scenario of increasing only senior level job opportunities by 20% throughout London. Senior level occupations are defined as non-routine occupations as coded in the LTDS, which include ‘modern professional occupations’, ‘senior managers or administrators’, ‘middle or junior managers’ and ‘traditional professional occupations’. Hence, this scenario corresponds to a policy encouraging only selected industries which employ such occupations.

**Fig. 10:**
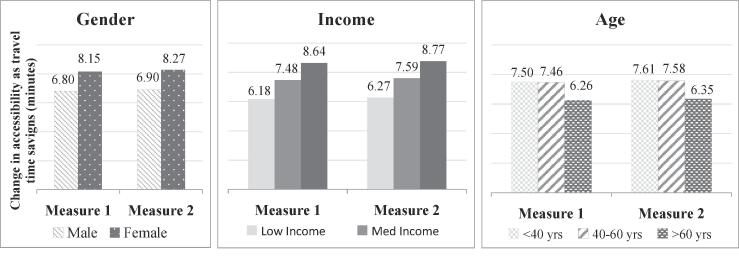
Accessibility impacts of land-use policy 2.

Compared to Policy 1, Policy 2 has a much higher impact on all population segments, which is expected as it involves a larger increase (20% as opposed to 10%) in jobs (Fig. 10). The impact of this policy is more pronounced for females compared to males, for high income compared to low income, and for young and medium age groups compared to older persons. Between the two measures, in this case they provide identical trends in accessibility, implying that the differences in accessibility levels across socio-demographic segments is not a result of differing abilities/preferences as reflected by individual characteristics.

#### 4.4.3 Policy 3: An increase in train fares by 20%

**Fig. 11:**
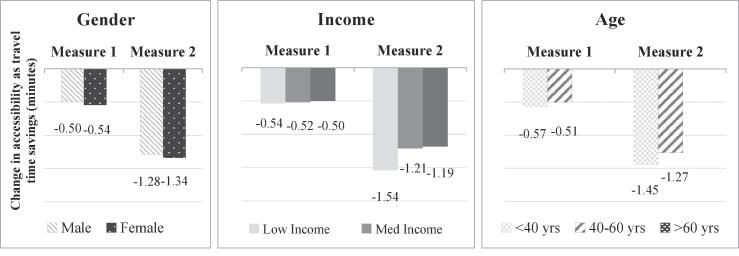
Accessibility impacts of transport policy 3.

For our third and fourth policy scenarios, we evaluate the equity impact of transport policies, starting with an increase in train fares of 20%. As anticipated, this policy results in a decrease in accessibility levels for all segments of the population (Fig. 11). We note that logsum measure 1 on average underestimates the impact of policy 3 for all sub-sections of the population. The reason for this is the higher travel cost sensitivity as captured by the mode-destination choice model from which logsum measure 2 is derived.

Regarding the difference in trends between the two logsum measures, we see that individual characteristics capture a higher impact of this policy on low income groups, which is not captured using measure 1 – this arises from the higher sensitivity to train cost for this segment. For all the other segments, the impacts show similar trends using both measures. Older persons are not affected by this (fare) policy as they are eligible for free travel by train.

#### 4.4.4 Policy 4: A decrease in bus density and increase in train density

**Fig. 12:**
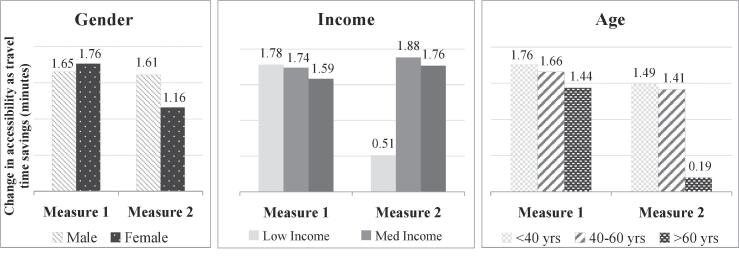
Accessibility impacts of transport policy 4.

The second transport policy was implemented by increasing the total bus travel times by 20% (to represent a decrease in bus network density) and decreasing the total train travel times by 20% (to represent an increase in train network density). The results of this analysis are shown in Fig. 12.

The aggregate impact of this policy is found to be positive for all population groups. This is because, as seen in Fig. 5, the accessibility by train is valued much higher compared to the accessibility by bus. In terms of trends by socio-demographic groups, contrasting results are obtained using the two measures. For example the distribution by gender shows that if individual characteristics are not considered, this policy is seen to have a higher positive impact on females compared to males (as demonstrated by the higher travel time savings in Fig. 12). But the individual abilities and preferences render the impact on females considerably lower (travel time savings drop from 1.76 min to 1.16 min), which is captured by measure 2. This is attributable to the higher preference females have for bus usage compared to males, and hence the reduction in bus accessibility is expected to impact females more. Similarly, ignoring individual characteristics also overestimates the impact of this policy on the low income group (who are much less sensitive to bus travel time) and older people (who have an inherent dis-preference for train).

#### 4.4.5 Discussion

Interesting conclusions emerge from the policy analyses. In the case of land-use policies both logsum measures 1 and 2 show the same trends suggesting that the individual characteristics do not significantly impact the predicted accessibility changes. On the other hand, the analysis of the transport policies are in agreement with the equity analysis carried out in Section 4.3 – logsum measure 1 often fails to capture important differences in accessibility arising due to personal characteristics, which are captured by logsum measure 2.

It is important to remember that the ability of a logsum measure to capture accessibility differences is strongly related to the model structure used for developing the logsum measure. The destination attractiveness term (number of opportunities specific to the occupation types) for the logsum measures used in this study is not segmented by socio-demographic categories and hence it cannot capture the differences arising due to individual characteristics. Hence, based on the findings of the current study, it is not possible to pass a conclusive judgment on the value of including individual characteristics in logsum measures of accessibility when evaluating land-use policies. Whereas the value of including individual characteristics in logsum measures of accessibility when evaluating transport policies is clearly demonstrated.

In the US, federal anti-discrimination law requires metropolitan planning organizations to conduct an equity analysis, with a focus on addressing disparate impacts. Several studies have highlighted the need for a more rigorous analysis of impacts of transport policies on minority communities, but clear guidance and methodology for such analysis is missing (Karner and Niemeier, 2013; Sanchez et al., 2004). Disparate impacts could be unintentional (Belton, 2005), with the same policy resulting in disproportionate burden to some communities. In order to be able to accurately capture such disparate impacts on accessibility, it is essential that the measure used is sensitive to the population demographics of interest. Our analysis in this paper highlights the fact that ignoring individual needs and constraints in logsum accessibility measures can mask the impact of such disproportionate burdens. Logsum measures with individual characteristics such as those used in our study could inform analysis of disparate impacts to fulfil equitable planning goals.

## 5 Conclusion

This paper explores the applicability of logsum measures to transport equity analysis. More specifically, it examines the impact of a logsum formulation including individual characteristics on the effectiveness of the equity analysis. We undertook a comprehensive assessment of equity that included spatial, social and economic equity analysis. The impact of the logsum measures on equity analysis for hypothetical policy scenarios was also conducted. The results of the analyses in this paper support the general finding in the literature (Litman, 2002; Talen and Anselin, 1998) that the outcome of the equity analysis can vary a lot depending on the indicator used. Moreover, our results demonstrate quantitatively that ignoring individual characteristics when developing logsum measures of accessibility can lead to unreliable equity analyses.

For spatial equity analysis, we observe that the variations within the population are typically averaged out at the zonal level and limited additional insight is derived by including individual characteristics. The real strength of using logsum measures of accessibility lies in their application to social and economic equity analysis. Our case study demonstrates that these measures can help identify the vulnerable groups and their issues far better than most available measures. The Gini index, as well as the socio-demographic trends – all suggest that ignoring individual characteristics in the logsum measure leads to a more equitable picture of accessibility across subsections of the population (males versus females, low versus medium and high incomes, young versus old). Effectively, logsum measures of accessibility that do not include sociodemographic characteristics fail to capture the variations in accessibility resulting from individual needs and abilities. Although there is no clear answer as to degree of the impact of including individual characteristics, it is important to be aware of the implications of excluding them, especially when the logsum measure is used for equity analysis.

Several assumptions had to be made for the analysis presented in this study, and the results are therefore limited by these assumptions. In terms of the model specifications used for the logsum measures, a limited number of variables were considered. Several details like parking charges, access/egress times, physical disability, etc. that might be important from an accessibility perspective were not available, and hence, could not be used. A more detailed model would likely be able to provide further insights into these issues. Another simplification in our analysis is ignoring the temporal variation in calculation of travel times, which is also a data limitation. However, incorporating temporal variation of travel times is expected to improve the models and affect different segments of the population differentially. So this is likely to further accentuate our findings related to the inclusion of individual characteristics. Finally, the joint mode-destination choice models used in this study presume equal substitutability for mode and destination, which might not always be true. Niemeier (1997) argues that while for a low income person, who is more likely to switch jobs, the assumption of joint mode-destination choice is reasonable; a high income person with a stable job might switch modes more frequently than the job. In extending the results of this paper to real-life application, it will therefore be necessary to explore several other model formulations.

## Footnotes

1 The standard deviation of *ε* is inversely related to the estimated parameters of the observed component of utility, and hence larger the scale factor *μ*, smaller the parameter estimates.

2 The validity of the travel time estimates is based on verification carried out by a working group made up of some UK councils and private sector companies (S. Court, personal communication, September 11, 2014). We undertook additional validation by comparing selected travel time estimates from TRACC with the travel times to chosen destination in the LTDS.

3 An income per person of less than £1000 per month is defined as low income, between £1000 to £2000 as medium income and greater than £2000 per person per month is classified as high income household.

4 It should be noted here that Gini coefficient is a measure of equality as opposed to equity. Equity refers to fairness, whereas equality refers to equal distribution of the concerned resource (van Wee and Geurs, 2011). While equity measurement depends on the value system being followed, most transport equity analysis measure equality which is more objective, and can be calculated directly (Carleton and Porter, 2018). Equality measurement can then serve as in input for equitable planning.
